# Polycystic ovary syndrome and risk of endometrial, ovarian, and breast cancer: a systematic review

**DOI:** 10.1186/s40738-016-0029-2

**Published:** 2016-12-05

**Authors:** Holly R. Harris, Kathryn L. Terry

**Affiliations:** 1 Program in Epidemiology, Division of Public Health Sciences, Fred Hutchinson Cancer Research Center, M4-859 Seattle, WA USA; 2 Department of Obstetrics and Gynecology, Brigham and Women’s Hospital and Harvard Medical School, Boston, MA USA; 3 Department of Epidemiology, Harvard T.H. Chan School of Public Health, Boston, MA USA

**Keywords:** Polycystic ovary syndrome, Endometrial cancer, Ovarian cancer, Breast cancer

## Abstract

**Background:**

Polycystic ovary syndrome (PCOS) is a complex endocrine disorder with an estimated prevalence of 4–21% in reproductive aged women. The altered metabolic and hormonal environment among women with PCOS may increase their risk of some types of cancer.

**Methods:**

We performed a comprehensive review of the literature using numerous search terms for all studies examining the associations between polycystic ovary syndrome and related characteristics and cancer published in English through October 2016. This review summarizes the epidemiological findings on the associations between PCOS and endometrial, ovarian, and breast cancers and discusses the methodological issues, complexities, and underlying mechanisms of these associations.

**Results:**

We identified 11 individual studies and 3 meta-analyses on the associations between PCOS and endometrial cancer, 8 studies and 1 meta-analysis for ovarian cancer, and 10 studies and 1 meta-analysis for breast cancer. Multiple studies reported that women with PCOS were at a higher risk for endometrial cancer; however, many did not take into account body mass index (BMI), a strong and well-established risk factor for endometrial cancer. The association with ovarian cancer was less clear, but a potentially increased risk of the borderline serous subtype was reported by two studies. No consistent association between PCOS risk and breast cancer was observed.

**Conclusion:**

The associations between PCOS and endometrial, ovarian, and breast cancer are complex, with the need to consider many methodological issues in future analyses. Larger well-designed studies, or pooled analyses, may help clarify these complex associations.

## Background

Polycystic ovary syndrome (PCOS) is a complex endocrine disorder characterized by oligomenorrhea, hyperandrogenism, and polycystic ovaries. It has an estimated prevalence of 4–21% in reproductive aged women depending on the diagnostic criteria and population examined [1–7]. In the U.S. it has been estimated that 5 million women may have PCOS [8]. PCOS is associated with metabolic abnormalities including dyslipidemia, insulin resistance, and type II diabetes and is also one of the most common causes of reduced fertility [9, 10]. The altered metabolic and hormonal environment among women with PCOS may increase their risk of some types of cancer.

First described by Stein and Leventhal in 1935 as women with polycystic ovaries, amenorrhea, and hirsutism [11], PCOS is currently clinically identified by one of three diagnostic criteria set by the National Institutes of Health/National Institute of Child Health and Human Disease (NIH/NICHD),[12] European Society for Human Reproduction and Embryology/American Society for Reproductive Medicine (ESHRE/ASRM) (Rotterdam criteria),[13] and Androgen Excess and PCOS Society [14]. While the definitions are similar with regard to assessment of menstrual history and androgen excess (defined by clinical characteristics or serum androgen levels) they differ in that the NIH definition does not consider ovarian morphology (Table 1). Thus, the prevalence of PCOS varies depending on the diagnostic criteria used with the NIH/NICHD criteria resulting in the lowest prevalence estimates followed by the Androgen Excess Society and ESHRE/ASRM [3].

**Table 1:** Diagnostic criteria for PCOS

	NIH 1990 Criteria	Rotterdam Criteria	AE-PCOS Society Criteria
Hyperandrogenism	Required	2 out of 3 required	Required
Ovulatory dysfunction (oligo- or amenorrhea)	Required	2 out of 3 required	Either ovulatory dysfunction or PCOS morphology required
PCOS morphology	Not required	2 out of 3 required	Either ovulatory dysfunction or PCOS morphology required

The clinical presentation of PCOS varies but commonly includes ovulatory dysfunction (oligomenorrhea, irregular periods and/or anovulatory cycles), hirsutism, acne, and polycystic ovaries (PCO). Women with PCOS commonly have insulin resistance and metabolic syndrome [15–19]. In addition, they are also at increased risk of type II diabetes,[20, 21] high cholesterol,[22–25], high blood pressure,[26–28] and are more likely to be overweight or obese [29–31]. Pharmacological agents are the first- and second-line treatments for many PCOS symptoms including: oral contraceptive use for oligomenorrhea, hirsutism, and acne; metformin for anovulatory infertility, oligomenorrhea, and type II diabetes prevention and treatment; and ovulation induction drugs (clomiphene and letrozole) for anovulatory infertility in this population [32].

### Epidemiologic evidence

#### Endometrial cancer

An association between PCOS and endometrial cancer was first suggested over fifty years ago by a series of small case reports, but these studies were limited by lack of control groups and small numbers [33]. Three meta-analyses, with overlapping studies, have reported a significant increased risk of endometrial cancer among women with PCOS (Table 2) [34–36]. However, they each included estimates from analyses that did not take into account BMI which is a common characteristic of PCOS and a strong and well-established risk factor for endometrial cancer [37, 38].

**Table 2:** Studies of PCOS and endometrial cancer risk

Author	Year	Study design	Endometrial Cancer Cases (n)	Overall result RR (95% CI)
Gottschau	2015	Registry cohort (Denmark)	16	3.9 (2.2–6.3)^a^
Shen	2015	Registry cohort (Taiwan)	7	4.7 (1.6–14.1)^a^
Barry^b^	2014	Meta-analysis	1264	2.8 (1.3–6.0)^a^
Haoula^c^	2012	Meta-analysis	938	2.9 (1.5–5.5)^a^
Brinton^d^	2010	Cohort (US)	15	2.0 (1.1–3.3)^a^
Fearnley^e^	2010	Case control (Australia)	156	2.2 (0.9–5.7)
Chittenden^f^	2009	Meta-analysis	667	2.7 (1.0–7.3)^c^
Zucchetto	2009	Case–control (Italy)	454	1.3 (0.7–2.2)
Iatrakis^e^	2006	Case–control (Greece)	81	9.0 (0.5–176.0)^a,g^
Pillay^h^	2006	Cross-sectional (UK)	128	1.0 (0.4–2.7)^a,c^
Niwa	2000	Case–control (Japan)	136	8.9 (0.4–184.9)^a,g^
Wild	2000	Cohort (UK)	11	6.1 (1.0–36.9)
Escobedo	1991	Case–control (US)	437	5.4 (2.4–12.3)^a^
Coulam^i^	1983	Cohort (US)	5	3.1 (1.1–7.3)^a^

^a^ Effect estimate not adjusted for BMI

^b^ This meta-analysis includes the studies by Fearnley, Zucchetto, Iatrakis, Niwa, and Escobedo

^c^ This meta-analysis includes the studies by Fearnley, Iatrakis, Pillay, Niwa, and Escobedo

^d^ In this study, androgen excess or menstrual disorders was evaluated rather than PCOS

^e^ This study only included women less than 50 years old

^f^ This meta-analysis includes the studies by Iatrakis, Pillay, Niwa, and Escobedo

^g^ Odds ratio was calculated based on numbers provided in the paper

^h^ In this study, polycystic ovary morphology was evaluated rather than PCOS

^i^ In this study, chronic anovulation syndrome was evaluated rather than PCOS

Five studies have examined the association between PCOS and endometrial cancer without accounting for BMI (Table 2). Two of these were registry-based cohorts reporting estimates of 3.9 (95% confidence interval [95% CI] = 2.2–6.3) [39] and 4.7 (95% CI = 1.6–14.1). Three case–control studies reported odds ratios (ORs) of 5.4 (95% CI = 2.4–12.3),[40] 8.9 (95% CI = 0.43–184.86),[41] and 9.0 (95% CI = 0.5–176.1) [42]. In addition, two studies examining chronic anovulation syndrome and androgen excess/menstrual disorders as proxies for PCOS, reported ORs of 3.1 (95% CI = 1.1–7.3) [43] and 2.0 (95% CI = 1.1–3.3),[44] respectively, while a study examining polycystic ovarian morphology reported no association (OR = 1.0; 95% CI = 0.4–2.7) [45]. Besides lack of adjustment for BMI these studies were additionally limited by a small number of exposed cases, consisting of 16 or fewer cases with PCOS. In studies that have reported effect estimates adjusted for BMI the observed associations with PCOS and endometrial cancer have been less consistent [46–48]. Wild et al. followed a cohort of women diagnosed with PCOS and reported an increased risk of endometrial cancer in these women compared to age-matched controls with OR of 5.3 (95% CI = 1.5–18.6) without adjustment for BMI and 6.1 (95% CI = 1.0–36.9) after adjustment for BMI [47]. In contrast, Fearnley et al. reported an attenuation of the OR following adjustment for BMI (OR = 4.0; 95% CI = 1.7–9.3 and OR = 2.2; 95% CI = 0.9–5.7, respectively) [46]. Finally, Zucchetto et al. observed no association between PCOS and endometrial cancer with a BMI-adjusted OR of 1.3 (95% CI = 0.7–2.2) with an OR that was slightly higher when limited to premenopausal women (OR = 2.0; 95% CI = 0.8–5.4) (Table 2) [48]. Differences in these studies may be due to varying control for confounders, Wild et al. did not adjust for any variables except BMI while Fearnley et al. adjusted for parity and oral contraceptive (OC) use, and Zucchetto et al. adjusted for parity, OC use, age at menarche, and age at menopause. In addition, the study population in Fearnley, et al. was limited to women under age 50 which is likely a primarily premenopausal population.

Endometrial tumors display a variety of histologic features but the majority of cases are adenocarcinomas (>95%) and can be classified into two main subtypes, endometrioid (Type I) and non-endometrioid (Type II) [49, 50]. As approximately 70–80% of endometrial cancers are Type I tumors the reported associations between PCOS and endometrial cancer are likely driven by this subtype. To our knowledge only one study has examined the association between PCOS and subtype specific endometrial cancer reporting a slightly stronger association between PCOS and endometrial cancer when limited to Type I (OR all cases = 2.2; 95% CI 0.9–5.7 vs. OR Type I cases = 2.4; 95% CI = 1.0–6.2) [46].

#### Ovarian cancer

PCOS has been hypothesized to increase ovarian cancer risk through increased androgen exposure [51]. Evidence linking androgens to ovarian cancer includes the presence of androgen receptors on normal ovarian cells as well as benign and borderline tumors, and a doubling of androgen levels during pregnancy is associated with a 40–50% increased risk of borderline serous and invasive mucinous tumors [51–53]. However, studies of prediagnostic androgen levels not during pregnancy have been mixed [54–57]. With respect to PCOS specifically, nine studies have examined the association with ovarian cancer risk to date [39, 43, 44, 58–63]. Many of these studies had limited power, with four studies having 12 or fewer ovarian cancer cases (Table 3) [39, 43, 44, 60, 62]. Overall, studies of PCOS and ovarian cancer have been largely null with some suggestion of an increased risk as demonstrated by a recent meta-analysis of three studies (OR = 1.4; 95% CI = 0.9–2.2) [34]. Since then a Danish registry study reported a non-significant increase in ovarian cancer risk for women with PCOS compared to the general Danish female population (standardized incidence ratio [SIR] = 1.8; 95% CI = 0.8–3.2), but the study was limited by small numbers with only 10 ovarian cases [39]. In a recent Taiwanese study with only 11 ovarian cancer cases, no association between PCOS and ovarian cancer was observed (hazard ratio [HR] = 1.0; 95% CI = 0.2–4.6) [62]. In the New England Case–control (NECC) study, which includes 1,513 ovarian cancer cases, we observed no association between self-reported PCOS and ovarian cancer risk (OR = 1.0; 95% CI = 0.6–1.5) [63].

**Table 3:** Studies of PCOS and ovarian cancer risk

Author	Year	Study design	Ovarian Cancer Cases (n)	Overall result RR (95% CI)
Harris	2016	Case–control (US)	1513	1.0 (0.60–1.5)
Gottschau	2015	Registry Cohort (Danish)	10	1.8 (0.8–3.2)^a^
Shen	2015	Registry Cohort (Taiwan)	11	1.0 (0.2–4.6)^a^
Barry^b^	2014	Meta-analysis	3363	1.4 (0.9–2.2)^a^
Bodmer	2011	Case–control (UK)	1611	1.6 (0.7–4.1)^a^
Brinton^c^	2010	Cohort (US)	12	1.8 (0.9–3.1)^a^
Olsen	2008	Case–control (Australia)	1276	1.1 (0.6–2.0)
Schildkraut	1996	Case–control (US)	476	2.4 (1.0–5.9)^a^
Rossing	1994	Cohort (US)	11	2.4 (0.2–22.5)

^a^ Effect estimate not adjusted for BMI

^b^ This meta-analysis includes the studies by Bodmer, Olsen, and Schildkraut

^c^ In this study, androgen excess or menstrual disorders was evaluated rather than PCOS

Epithelial ovarian cancer consists of molecularly and etiologically distinct subgroups that can be separated into four main histologic subtypes [64–67]. Thus, an elevation in risk relevant to only certain subtypes of ovarian cancer could be missed if subgroup specific estimates of ovarian cancer are not calculated. Only two studies to date have examined the association by histologic subtype. Olsen, et al. reported an association between PCOS and the borderline serous subtype (OR = 2.5; 95% CI 1.0–6.1) and noted this association was strongest among women with a BMI ≥ 25 (OR = 3.0; 95% CI 1.2–7.5) [59]. Consistent with this result, we observed in the NECC study of ovarian cancer the suggestion of an increased risk of the borderline serous subtype for women with PCOS (OR = 1.2; 95% CI 0.5–2.8) that was also stronger among overweight women [63]. Serous borderline ovarian tumors have been proposed to arise from benign ovarian tumors [68] and have higher androgen receptor levels than serous invasive tumors [52]. Furthermore, in a large European prospective cohort study, prediagnostic androstenedione was shown to increase risk of low-grade tumors (OR = 1.99; 95% CI = 0.98–4.06) but decrease risk of high grade tumors (OR = 0.75; 95% CI = 0.61–0.93) [57]. Larger studies are needed to further examine whether PCOS is associated with serous borderline tumors and whether this association only pertains to overweight women.

#### Breast cancer

Characteristics and consequences of PCOS have been previously associated with both increased and decreased risk of breast cancer. For example, infertility due to an ovulatory disorder has been shown to decrease breast cancer risk [69] while obesity increases breast cancer risk among postmenopausal women and decreases risk among premenopausal women [38]. However, studies to date have not observed an association between PCOS and breast cancer risk (Table 4). A meta-analysis including two case–control studies (from Italy and Iran) and one cohort study (Iowa Women’s Health Study) reported no elevation in risk for women with PCOS (OR = 1.0; 95% CI = 0.6–1.4) [34]. Similarly, a recent Danish registry study showed no association between PCOS and breast cancer risk with 59 breast cases observed and 56 expected resulting in a standardized incidence ratio of 1.1 (95% CI = 0.8–1.4) [39] and a retrospective cohort study in Taiwan showed no association (HR = 1.6, 95% CI = 0.9–2.8) [62].

**Table 4:** Studies of PCOS and breast cancer risk

Author	Year	Study design	Breast Cancer Cases (n)	Overall result RR (95% CI)
Gottschau	2015	Registry Cohort (Danish)	59	1.1 (0.8–1.4)^a^
Shen	2015	Registry Cohort (Taiwan)	44	1.6 (0.9–2.8)^a^
Barry^b^	2014	Meta-analysis	3618	1.0 (0.6–1.4)^a^
Ghasemi	2010	Case–control (Iran)	166	0.7 (0.3–1.5)^a^
Brinton^c^	2010	Cohort (US)	89	1.3 (1.1–1.6)^a^
Terry^d^	2006	Cohort (US)	2267	0.8 (0.6–1.0)
Wild	2000	Cohort (UK)	49	1.3 (0.6–2.8)
Anderson	1997	Cohort (US)	883	1.2 (0.7–2)
Talamini	1997	Case–control (Italy)	2569	0.9 (0.4–1.8)
Gammon	1991	Case–control (US)	4730	0.47 (0.3–0.9)^a^
Coulam^e^	1983	Cohort (US)	12	1.5 (0.8–2.6)

^a^ Effect estimate not adjusted for BMI

^b^ This meta-analysis includes the studies by Ghasemi, Anderson, and Talamini

^c^ In this study, androgen excess or menstrual disorders was evaluated rather than PCOS

^d^ In this study, infertility due to ovulatory disorders was evaluated rather than PCOS

^e^ In this study, chronic anovulation syndrome was evaluated rather than PCOS

Though women were not specifically classified by PCOS status, we reported in the Nurses’ Health Study II, a cohort including more than 100,000 women and followed prospectively, that women who reported infertility due to ovulatory disorders had no increase in breast cancer risk. In fact, women with ovulatory disorders that were treated for infertility had a significantly lower risk compared to women with no reported infertility [69]. Anovulatory cycles skip the luteal phase of the menstrual cycle and it is during the luteal phase that estrogen and progesterone levels are both elevated [70]. In addition, breast cancer cell proliferation is higher during the luteal phase [71, 72] thus women with ovulatory disorders will have a reduced lifetime exposure to luteal phase hormones which may explain the observed reduction in risk.

#### Other cancers

The literature examining the association between PCOS and cancers other than endometrial, breast, and ovarian, is scarce. Although not previously described, more kidney, colon, and brain cancers were observed among women with PCOS compared to expected rates in the general Danish population [39]. In addition, Brinton et al. reported a significant standardized incidence ratio for melanoma (SIR = 2.0; 95% CI = 1.1–3.2) among infertile women with androgen excess or menstrual cycle disorders compared to the general U.S. population [44]. These findings need to be followed up in large well-designed studies for validation.

## Discussion

### Confounding and mediation

Since high BMI is a common characteristic of PCOS, [29–31] one cannot exclude the possibility that reported associations may be attributable to a higher BMI in women with PCOS, on average. As noted above, many studies failed to control for BMI and when BMI is taken into account residual confounding may remain since weight is strongly associated with these diseases. In fact, the inverse association between BMI and breast cancer among premenopausal women but positive association among postmenopausal women may explain, in part, the lack of an association with breast cancer [38]. Conversely, there is evidence that PCOS influences body size through dietary intake and cravings,[73, 74] as well as through its influence on metabolic factors that may impact weight gain [75–81]. Thus BMI may be both a mediator and confounder of the PCOS and cancer associations making it difficult to characterize a BMI-independent PCOS association.

Beyond BMI, the adjustment for cancer risk factors varied widely between studies and may also explain differences between studies as some characteristics of PCOS, such as infertility, are risk factors for these cancers. Reproductive risk factors such as parity and age at first birth may have different distributions in those with and without PCOS due to the increased risk of infertility in women with PCOS. In addition, women with PCOS are at increased risk of type II diabetes [20, 21], insulin resistance, and metabolic syndrome [15–19]. These conditions have been associated with cancer risk,[82, 83] and thus could serve either as potential confounders or intermediates, of the PCOS-cancer association. A few studies have adjusted for these potential intermediate variables (i.e. hypertension and diabetes), which may have resulted in an attenuation of the true association [46, 62].

### Possible biologic mechanisms

PCOS is characterized by prolonged anovulation with consequent exposure to estrogen unopposed by progesterone [33, 84], which may explain why women with PCOS have an elevated risk of endometrial cancer, an estrogen-sensitive disease. The association between estrogen and breast or ovarian cancer are more complex and depend on subtypes of disease and menopausal status,[65, 66, 85, 86] which may explain the lack of overall increased risk for these cancers. Larger studies, or pooled analyses, are needed which would allow investigation of these associations by specific subtypes and menopausal status.

Alternatively, elevated androgens may play a role. Hyperandrogenism is one major component of all three clinical diagnostic criteria for PCOS. In 1998, Risch proposed that ovarian cancer might be associated with factors related to androgen stimulation of ovarian cells,[51] and animal studies have demonstrated that testosterone stimulates the growth of epithelial cells in the ovary [87]. The suggestion of an increased risk of the borderline serous subtype of ovarian cancer among women with PCOS supports the hypothesis that androgens could be a factor in ovarian cancer development as androgen receptors have been shown to be higher in serous borderline tumors compared to serous invasive [52]. Circulating androgens may also play a role in breast carcinogenesis [88]. In a pooled analysis of prospective studies, risk of breast cancer has been shown increase with increasing testosterone levels in postmenopausal women [89]. A similar increased risk has been reported by cohort studies among premenopausal women [90, 91].

### PCOS medications that may influence cancer risk

#### Oral contraceptives

For women who are not attempting to become pregnant, combined oral contraceptives are often one of the first-line treatments for menstrual irregularities in women with PCOS [92]. Combined oral contraceptives are associated with decreased risk of ovarian [93] and endometrial cancers [94]. The protective effect of oral contraceptives on ovarian cancer risk is likely explained by a decreased lifetime number of potentially damaging ovulations,[95] while the protective effect in endometrial cancer may be the result of reducing exposure to unopposed estrogen which limits the cell proliferation that is stimulated by estrogens [84]. In contrast, oral contraceptives may result in a very small short-term increase in breast cancer risk, while 10 years after cessation of use the risk among women who had used oral contraceptives was similar to those who had not used oral contraceptives [96].

#### Metformin

Among women with PCOS, metformin is prescribed to treat or reduce the risk of type II diabetes, improve insulin resistance, and in some cases to help regulate the menstrual cycle/induce ovulation. Evidence has suggested that metformin use may be protective against various forms of cancer. This is supported by laboratory studies that demonstrate metformin’s anti-cancer activities [97]. However, fewer studies have examined the impact of metformin on incidence of endometrial, breast, and ovarian cancer.

Breast cancer has been the most studied of the three cancers with respect to the influence of metformin. A 2014 meta-analysis including 13 studies reported a summary risk estimate (SRR) of 0.9 (95% CI = 0.8–1.0) for the association between metformin use and breast cancer incidence [98]. When limited to the studies which adjusted for BMI (*n* = 7), an established risk factor for postmenopausal breast cancer, the SRR was 0.8 (95% CI = 0.7–1.0), and when limited to prospective studies (*n* = 7), the SRR was 0.9 (95% CI = 0.9–1.0). However, a more recent analyses among women in general practice databases from Germany and the United Kingdom reported no association between metformin users and breast cancer incidence when compared users of sulfonylurea and insulin (HR = 1.0; 95% CI = 0.8–1.3 and HR = 1.1; 95% CI = 0.7–1.7, respectively) [99].

The epidemiologic data examining the relation between metformin and endometrial cancer have generally suggested no association or a protective association between metformin and endometrial cancer. Among women in the UK-based General Practice Research Database (GPRD) no significant association was observed between metformin and endometrial cancer with an OR of 0.9 (95% CI = 0.6–1.2) [100] Similarly, in a retrospective cohort analysis of US healthcare claims data no association was observed between metformin use and endometrial cancer (HR = 1.1; 95% CI = 0.9–1.4) [101]. Most recently, Tseng reported an inverse association between metformin and endometrial cancer among women with type II diabetes in the National Health Insurance database in Taiwan (HR = 0.7; 95% CI = 0.6–0.7) [102].

The association between metformin use and ovarian cancer has been less studied than breast or endometrial cancer. However, metformin use has been demonstrated to influence ovarian cancer cell growth in vitro and in vivo [103, 104] and has been associated with survival in patients with ovarian cancer [105, 106]. To our knowledge, only one observational study has reported the association between metformin and risk of ovarian cancer observing the suggestion of a decreased risk of ovarian cancer with increasing metformin use (OR = 0.6; 95% CI = 0.3–1.3) among women in the UK-based GPRD [58].

#### Ovulation-induction therapies

Previous studies on the associations between fertility drugs and risk of hormone related cancers have produced mixed results. The inconsistencies are likely due to the combining of different drug types, self-reported drugs use, limited power and information about dosages, short follow-up, and reproductive confounders that are highly correlated with drug use [107]. Clomiphene (clomid), a common treatment for PCOS-associated infertility, is a selective estrogen receptor modulator that stimulates the ovaries to ovulate but can increase or decrease estrogen receptor activation in other tissues. In breast cancer cell lines, clomiphene has a pro-apoptotic effect,[108] which may explain why women who received clomiphene for PCOS-related infertility have a reduced breast cancer risk in some studies [69, 109]. A more recent study in a cohort of over 12,000 infertile women reported that clomiphene was not associated with breast cancer risk, although a non-significant increased risk was observed for those with over 12 cycles [110].

Clomiphene’s action as an ovulation inducer provides strong biological plausibility for its influence on ovarian cancer risk as ovulatory damage likely plays a prominent role in ovarian cancer risk [95]. Most recent studies have indicated no significant increased risk of ovarian cancer among clomiphene users [107]. In a large cohort of women evaluated for infertility with extended follow-up Trabert, et al. reported no overall association between clomiphene use and ovarian cancer risk (HR = 1.3; 95% CI = 0.9–2.1), however they did note an increased risk among women who remained infertile (HR = 3.6; 95% 1.4–9.7) [111] and this increased risk in nulligravid women has been observed in other studies [107]. Whether this indicates confounding by infertility severity or a true effect deserves further study.

Endometrial cancer has been the least studied of the hormone related cancers in relation to clomiphene use. An association has been hypothesized since clomiphene has chemical properties similar to tamoxifen, a drug that has been associated with endometrial cancer risk [112]. However, most previous studies of clomiphene and endometrial cancer have been limited by power [107]. In one of the largest studies to date Brinton et al. reported a slight non-significant increase in risk of endometrial cancer among clomiphene users (HR = 1.4; 95% CI = 1.0–2.0) [113].

Fewer studies have examined the associations between clomiphene and cancers other then endometrial, ovarian, or breast cancer. A recent analysis in a cohort of over 12,000 infertile women reported that clomiphene was statistically significant increased risk of melanoma (HR = 2.0; 95% CI = 1.2–3.2), a non-significant increased risk of thyroid cancer (1.6; 95% CI = 0.9–2.8), and no increased risks for colorectal or lung cancer [114].

Letrozole, an aromatase inhibitor, has more recently been considered as an additional treatment option for anovulatory infertility. While longitudinal data does not yet exist to adequately examine the association between letrozole use for ovulatory infertility and hormone related cancer risk, letrozole is currently used as an adjuvant treatment for hormone receptor positive postmenopausal breast cancer [115] thus it could be hypothesized that it would likely decrease hormonal related cancer risk.

## Conclusions

The associations between PCOS and endometrial, ovarian, and breast cancer are complex, requiring consideration of PCOS diagnostic criteria, etiologic heterogeneity of cancer subtypes, confounding and mediating factors, menopausal status, co-morbid conditions, as well as treatment options that may also influence cancer risk. In addition, the rarity of ovarian and endometrial cancers make these cancers even more difficult to study. Larger well-designed studies, or pooled analyses, may help clarify these complex associations.

## Abbreviations

BMI: Body mass index
CI: Confidence interval
ESHRE/ASRM: European society for human reproduction and embryology/American society for reproductive medicine
GPRD: General practice research database
HR: Hazard ratio
NECC: New England case–control
NIH/NICHD: National institutes of health/national institute of child health and human disease
OR: Odds ratio
PCO: Polycystic ovaries
PCOS: Polycystic ovary syndrome
SIR: Standardized incidence ratio
SRR: Summary risk estimate
